# Differences in per capita rates of revascularization and in choice of revascularization procedure for eleven states

**DOI:** 10.1186/1472-6963-6-35

**Published:** 2006-03-16

**Authors:** Edward L Hannan, Chuntao Wu, Mark R Chassin

**Affiliations:** 1 School of Public Health, University at Albany, One University Place, Rensselaer, NY 12144, USA; 2 Mount Sinai School of Medicine, Box 1077, One Gustave L. Levy Place, New York, NY 10029, USA

## Abstract

**Background:**

A few studies have investigated differences in elective procedure rates across small and medium sized referral regions. The purposes of this study are to investigate differences in revascularizations across 11 entire states and to investigate differences in choice of revascularization procedure (percutaneous coronary intervention (PCI) vs. coronary artery bypass graft (CABG) surgery).

**Methods:**

Age-sex adjusted rates per 100,000 population who were 20 or older were calculated for PCI, CABG surgery, and total revascularization for each state. Also, the risk-adjusted proportion of revascularized patients who underwent PCI was calculated for each state and differences were compared.

**Results:**

We found variations in procedures performed per capita of 1.83-fold for PCI, 1.54-fold for CABG surgery, and 1.54-fold for total revascularization. For patients undergoing revascularization of two or more vessels, the age/sex adjusted maximum rate of 224 per 100,000 population over 20 years old in Florida was 53% higher than the minimum rate of 146 in Colorado. Higher catheterization rates per 1,000 Medicare enrollees and higher percent of white patients were significant predictors of higher revascularization rates, and density of specialists was a significant predictor of catheterization rate. The risk-adjusted percentage of revascularized patients with two or more arteries attempted who underwent PCI ranged from 10.4% in Oregon to 29.0% in Iowa.

**Conclusion:**

There are reasonably large differences among states in total revascularization rates and in type of revascularization among revascularization. These differences appear to be related to practice pattern differences. Future effort should be devoted to understanding the reason for these differences and the impact on patients' health and survival.

## Background

Some of the most notable and early investigations of healthcare practice pattern variations were a series of studies that demonstrated that the per capita rates of common elective procedures and the rates of hospitalization vary widely across small geographic regions [1-4]. Although these findings reflected care in the United States many years ago, there is evidence that the same magnitude of variation exists in more recent years.  Birkmeyer et al. found that for Medicare patients in 1995, lower extremity revascularization, carotid endarterectomy, back surgery, and radical prostatectomy rates had particularly high variations, and coronary artery bypass grafting (CABG), transurethral prostatectomy, mastectomy, and total hip replacement also had high variation in rates across regions [5].

With respect to the explanation for differences in the number of cardiac procedures performed per capita, studies in Canada [6] and the United Kingdom [7] have noted large variations in revascularization (CABG surgery and percutaneous coronary interventions (PCIs)), and data from the Organisation for Economic Co-Operation and Development show large differences in the cardiac surgery facilities per capita [8]. In America, recent studies in northern New England have found differences in revascularization rates per capita that are related to coronary angiography rates per capita [9] and to catheterization laboratories per capita [10]. The variation in revascularization rates was not found to be related to cardiologist supply [9]. Also, Hannan and Kumar found large differences in revascularization rates across 12 regions in New York State [11].

Another interesting question related to variation in cardiac procedures is whether there is a substitution effect. That is, do regions with low rates of CABG surgery have high rates of PCI because there is a difference of opinion across regions with regard to which procedure is appropriate for certain types of patients? Or do regions with high CABG surgery rates also have high PCI rates because high rates are related to other factors such as supply of specialists performing the procedures or the rate of diagnostic procedures (cardiac catheterization) being performed? One study of this question found a relatively high correlation (R = .49) between the CABG rate and the PCI across 305 Metropolitan Statistical Areas [12].

The purposes of this study are to investigate differences in revascularizations across 11 entire states and to investigate differences in choice of revascularization procedure (PCI vs. CABG surgery).

## Methods

### Data and patients

Data in the study came from the Agency for Healthcare Research and Quality's Healthcare Cost and Utilization Project (HCUP). The information is derived from 1999 administrative data that includes patient demographics, diagnoses, procedures performed, and discharge information for all inpatients in numerous states. We chose eleven states (Arizona, California, Colorado, Florida, Iowa, Maryland, New York, Oregon, South Carolina, Washington, and Wisconsin) in an attempt to achieve geographical balance.

Patients of interest were all patients who were at least 20 years old who underwent either isolated (meaning no other major cardiac procedures performed during the same admission) CABG surgery or PCIs as a primary procedure in one of these eleven states and were discharged in 1999.

### Data analysis

Rates per 100,000 population who were 20 and older were calculated for age (<40, 40–49, 50–59, 60–69, 70–79, and ≥ 80) and sex. Age/sex directly standardized rates of PCI, CABG surgery, and revascularization (PCI and/or CABG surgery) per 100,000 who were 20 and older were calculated for each state, using all 11 states as the population for standardization. For each of the three procedures or combination of procedures, the ratio of each statewide rate to the overall rate per capita was calculated and tested for statistical significance using the formula for the standard error of the directly standardized rate [13]. These rates were then calculated separately for one coronary vessel attempted, and for two or more vessels attempted (available data did not enable us to separate patients with two and three vessels attempted). Also, the ratio of the highest to lowest statewide rate was calculated.

The risk-adjusted percentage of patients undergoing PCI among all revascularized patients was calculated by state for patients with one coronary vessel attempted, and for two or more coronary vessels attempted. A logistic regression model was used to predict the probability of PCI for each revascularized patient based on age, sex, type of admission (emergency, other), recent acute myocardial infarction, and comorbidities identified by Elixhauser et al. [14]. A total of 10 secondary diagnosis fields were examined to determine the presence of comorbidities because the number available varied by state and 10 was the minimum. For each state, the predicted number of revascularized patients who received PCI was calculated by summing the predicted probabilities for all patients. This number was then divided by the number of observed PCI patients in the state, and the resulting quotient was multiplied by the overall percentage of revascularized patients undergoing PCI across all 11 states to obtain a risk-adjusted percentage of PCI patients in each state.

The ratios of these state-level percentages of PCI patients to the overall percentage for all states were calculated along with ratios of highest to lowest state-level proportions. Race was not used in the risk-adjustment process because not all states reported data on race.

This exercise was then repeated after taking patients with recent acute myocardial infarctions (primary angioplasty patients) out of the database and risk-adjusting the proportions of patients undergoing PCI using age, sex, and the significant comorbidities determined earlier.

The density of specialists [15] (cardiologists, cardiac surgeons, general invasive cardiologists, interventional cardiologists), the rate of cardiac catheterizations [16], a set of surrogates for socio-economic status (per cent of population that is white [17], per cent of population below poverty level [17], per cent unemployed [17], and per cent Medicaid [18]), and measures of heart disease in the states calculated using the HCUP data (age/sex adjusted admissions/100,000 population age 20 or older for acute myocardial infarction, age/sex adjusted admissions/100,000 population age 20 or older for coronary heart disease) were correlated with statewide total revascularization rates in order to assess the impact of each of these measures of supply or socio-economic status on utilization. Also, a stepwise linear regression (with p < 0.05) was performed with statewide revascularization rate as the dependent variable and the various supply and socio-economic states measures mentioned above as independent variables.

## Results

Table 1 presents the revascularization rates per 100,000 who were 20 and older by sex and for six different age groups in 1999 for PCI, CABG surgery and the two revascularization procedures combined. As indicated, the overall rates for PCI and CABG surgery were 266 and 147 per 100,000, respectively. The overall rate for any revascularization was 409 per 100,000, slightly lower than the sum of these two rates because some patients underwent both procedures in the same stay.

For PCI, there was considerable state-to-state variation, with the maximum age/sex adjusted rate per 100,000 of 340 in Florida being 83% higher than the minimum rate of 186 in Oregon (see Table 2). The variation in age/sex adjusted rates for CABG surgery among the eleven states was also high, although not as high as it was for PCI. The maximum rate was 182 in Florida, which was 54% higher than the minimum rate of 118 in Colorado. When the two procedures were combined, the variation was similar to the variation for CABG surgery, with the maximum rate of 517 per 100,000 in Florida being 54% higher than the minimum rate of 335 in Oregon (Table 2).

The correlation in CABG surgery rates per 100,000 and PCI rates per 100,000 (not in tables) was positive and relatively high, although not statistically significant (R = 0.47, p = 0.14). When one state with a very low PCI rate (Oregon) was removed, the correlation was statistically significant (R = 0.64, p = .046).

When revascularization rates were limited to patients with one coronary vessel attempted (not in Table), the variation was higher than it was when the data were limited to patients with two or more coronary vessels attempted. For patients with one vessel attempted, the highest age/sex adjusted revascularization rate was 292 in Florida, which was 70% higher than the minimum rate of 172 in Oregon. For patients with two or more vessels attempted, the maximum rate of 224 in Florida was 53% higher than the minimum rate of 146 in Colorado.

Table 3 examines reasons for differences among states in revascularization rates by looking at the relationship between states' total revascularization rates and a set of surrogates for socio-economic status (% of population that is white, % of population below poverty level, and % Medicaid), the supply of specialists (numbers of cardiovascular surgeons, all cardiologists, general invasive cardiologists and interventional cardiologists per 100,000 population), the number of cardiac catheterizations per 1,000 Medicare enrollees, and measures of heart disease in the states (age/sex adjusted admissions/100,000 population age 20 or older for acute myocardial infarction, age/sex adjusted admissions/100,000 age 20 or older for coronary heart disease). As indicated, the only variables that were significantly correlated with revascularization rate for all patients were the number of cardiovascular surgeons per 100,000 population, the number of cardiac catheterizations per 1,000 Medicare enrollees, and the age/sex adjusted admission rate for coronary heart disease. When all independent variables in Table 3 were entered in a stepwise linear regression (p < 0.05) with revascularization rate as the dependent variable, the only significant independent variables were the % of population that is white and the number of cardiac catheterizations per 1,000 Medicare enrollees (not shown in Table). The R^2 ^statistic was .94, meaning that 94% of the variation in revascularization rates could be explained by these two variables. The cardiac catheterization rate explained 68% of this variation by itself. Significant independent predictors of catheterization rate were coronary heart disease admission rate and the sum of the number of cardiac surgeons and the number of interventional cardiologists per 100,000 population. These two factors explained 89% of the variation in cardiac catheterization rates.

Table 4 presents, by state, the age/sex adjusted percentage of patients revascularized (i.e., patients who underwent PCI or CABG surgery) that underwent PCI procedures. This is done for patients with one vessel attempted, and for two or more vessels attempted. For patients who underwent revascularization of one coronary artery, the vast majority (96.8%) underwent PCI. This percentage did not vary substantially among states, with a maximum of 97.9% in Maryland and a minimum of 94.0% in Arizona. The percentage of patients undergoing revascularization of two or more coronary vessels who underwent PCI varied considerably more. The overall percentage was 22.3%, with the maximum percentage of 29.0% in Iowa being 2.80 times the minimum percentage of 10.4% in Oregon.

When revascularizations for patients with recent acute myocardial infarctions were removed from the group of patients in Table 4, the percentage of patients with one coronary vessel attempted who underwent PCI was very high (96.1%), and there was consequently little state-to-state variation in age/sex adjusted percentages (not shown in Table). However, the variation for patients with two or more vessels attempted was even higher than it was among all patients who were revascularized. The maximum percentage of revascularized patients who underwent PCI was in Iowa (26.9%), which was more than 3 times as high as the percentage in Oregon (7.8%).

## Discussion

In the course of the last 30 years, there have been several studies, initially by Wennberg and associates, that have demonstrated large differences in the rates of elective procedures across small geographic regions and across relatively large geographic areas (e.g., 306 hospital referral regions across the United States) [1-11]. Some of the procedures for which these differences have been found include tonsillectomy, hysterectomy, prostatectomy, carotid endarterectomy, hip replacement, and coronary artery bypass graft surgery.

For example, for cardiac procedures, Hannan and Kumar [11] found more than a three-fold variation in age/sex adjusted CABG rates and more than a two-fold variation in age/sex adjusted PCI rates in 12 regions in New York State. Also, Wennberg et al. found that there 82% of the variation (p < .001) in revascularization rates across twelve service areas in northern New England could be explained by differences in coronary angiography rates [9], and in another study Wennberg et al. found that in the same regions 43% of the variation in revascularization rates could be explained by differences in the number of catheterization laboratories per capita [10].

Our study differs from previous studies in that the regions that were identified (states) were considerably larger than regions that were examined in previous studies, and therefore less likely to exhibit large variations in utilization rates. Also, unlike some studies, we used HCUP data because it allowed us to identify all patients in a region receiving a procedure, not just Medicare patients.

Findings of our study were that there were large variations across states in age/sex adjusted PCI, CABG surgery and revascularization rates, although the variations were not as large as those found in other studies, all of which used considerably smaller geographical regions. We found variations of 1.83-fold for PCI, 1.54-fold for CABG surgery, and 1.54-fold for revascularization across the eleven states of interest.

In an attempt to explain differences in revascularization rates among states, we examined the relationship between revascularization rates and a few factors: the concentration of relevant specialists, the utilization of diagnostic catheterizations, proxies for the concentration of coronary artery disease, and socio-economic status. When these factors were tested in a linear regression model with statewide revascularization rate as the dependent variable, the only significant independent predictors were catheterization rate (positive correlation, p < 0.0001) and per cent white (p = 0.005). These two variables explained 94% of the variation in statewide revascularizaton rates, and cardiac catheterization rate explained 68% of the variation by itself.

High correlations between revascularization rates and cardiac catheterization rates have been found in earlier studies [9]. This could be related to the fact that states with sicker patients need to have more catheterizations and more revascularizations done. However, another possible explanation is that there are practice pattern differences among states in performing cardiac catheterization and revascularization, and that the states in which more patients are catheterized, more patients will be identified as needing revascularization. The latter hypothesis would appear to account for at least some of the differences found given the magnitude of the differences. In fact, one of the significant predictors of catheterization rate was the sum of the number of cardiac surgeons and interventional cardiologists per 100,000 population. Thus, the hypothesis that higher rates of procedures are associated with the "enthusiasm" and density of specialists who perform those procedures^16 ^did appear to have some merit.

The significant relationship between white population and revascularization rate has been demonstrated in numerous studies conducted in smaller geographic regions than the regions examined in this study [20-27]. Our findings are disturbing because they suggest that minorities may have lower access to cardiac procedures after controlling as well as possible for need for these procedures, and that these access differences persist even across very large sub-populations of our country.

We also found a positive, although non-significant correlation between PCI and CABG surgery rates (R = 0.47, p = 0.14). However, when Oregon, which had a very low PCI rate, was removed, the correlation became statistically significant (R = 0.64, p = .046). Thus, there was not an observed tendency for substitution of procedures among states. These findings are similar to the findings of Kuhn et al., who reported that there was a .49 correlation between PCI and CABG surgery rates for Medicare patients across 305 Metropolitan Statistical Areas in 1988 [12]. These authors concluded that the rates were correlated because they were both highly correlated with the rates of cardiac catheterization (with respective correlations of .64 and .72), which was also the case in our study.

We also found that the proportion of patients who were revascularized who underwent PCI was quite variable across states. For patients with multiple vessels attempted, the risk-adjusted proportion who underwent PCI ranged from 10.4% in Oregon to 29.0% in Iowa. Both Oregon and Iowa had ratios that were more than 25% different than the eleven state mean.

There are a few caveats to the study. First, it is possible that differences in revascularization rates among states could be related to the differences in coronary heart disease, and therefore need for revascularization, rather than in practice pattern differences among states. Although we used age/sex adjusted acute myocardial infarction (AMI) admissions and coronary heart disease (CHD) admissions per 100,000 population to adjust for need for revascularization when examining the impact of race and catheterization rate on revascularization rate, they are both flawed when used for this purpose. For instance, many patients who need revascularization have not suffered AMIs. Also, many patients with CHD are not admitted to the hospital unless there is an intent to revascularize them, so hospitalizations for CHD are undercounts of the number of patients who need revascularization. They may also be overcounts because not all patients with CHD require revascularization. Also, the analyses in Table 3 that identified predictors of revascularization rate suffer from the use of ecological variables.

Second, identified differences between states in the choice of revascularization procedure were necessarily limited to inpatient data. Some PCIs are performed in an outpatient setting and the tendency for PCIs to be performed on an outpatient basis may vary between states. However, although many of the states studied do not have outpatient databases, it does not appear that many PCIs were performed outside of the inpatient setting in the year of our study (1999).

Third, we were limited to using administrative data in the study, and some data elements we would have liked to use were not available. In particular, it would have been desirable to have all data elements that are needed to determine the appropriateness of each procedure for purposes of the part of the study relating to choice of procedure, including the number of diseased coronary vessels. It is valuable to be able to distinguish between two-vessel disease and three-vessel disease because most of the latter group undergo CABG surgery. Nevertheless, one would expect that in regions as large as entire states, this type of bias would be minimized.

Thus, in conclusion, we found large inter-state differences in the rates of revascularization and in the tendency to choose revascularization procedures. As noted earlier, differences in regional procedure rates have been reported in several older studies and in some recent studies. Our study differs in that the regions we used (all patients in entire states) are considerably larger than regions used in earlier studies. Despite this fact we still found that there were substantial differences in the use of cardiovascular procedures. Other findings of the study suggest that these differences are due in part to access related to socio-economic status and to practice pattern differences. We look forward to other studies that examine variations in procedure choice and rates and to the development of databases that are capable of arriving at more definitive explanations for differences of this magnitude.

## Conclusion

There are reasonably large differences among states in total revascularization rates and in type of revascularization among revascularization. These differences appear to be related to practice pattern differences. Future effort should be devoted to understanding the reason for these differences and the impact on patients' health and survival.

## Competing interests

The author(s) declare that they have no competing interests.

## Authors' contributions

ELH conceived the study, participated in the design of the study, and drafted the manuscript.

CW participated in the design of the study, performed the statistical analyses, and helped to draft the manuscript.

MRC participated in the design of the study and helped to draft the manuscript.

All authors read and approved the final manuscript.

## Pre-publication history

The pre-publication history for this paper can be accessed here:



## Figures and Tables

**Table 1:** Revascularization rate (per 100,000 age 20 and older) by age and sex, 1999.

Sex		PCI		CABG		PCI and/or CABG	
				
	Age (years)	% of Cases	Rate	% of Cases	Rate	% of Cases	Rate
Male	< 40	2	18	1	4	2	22
	40–49	12	205	7	72	10	274
	50–59	25	619	22	321	24	931
	60–69	28	1,028	31	693	29	1,705
	70–79	25	1,253	31	930	28	2,165
	≥ 80	8	863	8	497	8	1,350
Female	< 40	1	4	1	2	1	6
	40–49	6	54	4	18	6	71
	50–59	17	193	15	83	16	273
	60–69	26	434	28	221	27	647
	70–79	34	652	39	347	36	989
	≥ 80	15	455	12	170	14	620
Male		67	366	72	218	68	579
Female		33	172	28	81	32	251
Total			266		147		409
No. of Revascularizations			198,042		109,818		304,961

**Table 2:** Age/sex adjusted revascularization rates (per 100,000 age 20 and older) by state, 1999.

	PCI			CABG			PCI and/or CABG		
			
State	No. of Cases	Rate	Ratio to Mean (95% CI)	No. of Cases	Rate	Ratio to Mean (95% CI)	No. of Cases	Rate	Ratio to Mean (95% CI)
AZ	10,576	287	1.08 (1.06, 1.10)	5,131	138	0.93 (0.91, 0.96)	15.520	420	1.03 (1.01, 1.04)
CA	50,436	239	0.90 (0.89, 0.91)	28,537	136	0.92 (0.91, 0.93)	78,099	371	0.91 (0.90, 0.91)
CO	6,932	261	0.98 (0.96, 1.00)	3,035	***118***	***0.80 (0.77, 0.83)***	9,856	374	0.91 (0.90, 0.93)
FL	47,423	***340***	***1.28 (1.27, 1.29)***	26,023	***182***	***1.23 (1.22, 1.25)***	72,713	***517***	***1.26 (1.25, 1.27)***
IA	7,411	326	1.22 (1.20, 1.25)	3,440	150	1.02 (0.99, 1.05)	10,796	474	1.16 (1.14, 1.18)
MD	9,307	257	0.97 (0.95, 0.99)	4,620	129	0.88 (0.85, 0.90)	13,779	382	0.93 (0.92, 0.95)
NY	33,429	245	0.92 (0.91, 0.93)	18,182	134	0.91 (0.90, 0.92)	51,352	377	0.92 (0.91, 0.93)
OR	4,716	***186***	***0.70 (0.68, 0.72)***	3,812	152	1.03 (1.00, 1.06)	8,452	***335***	***0.82 (0.80, 0.84)***
SC	8,053	281	1.06 (1.03, 1.08)	4,859	170	1.15 (1.12, 1.18)	12,804	447	1.09 (1.07, 1.11)
WA	8,765	218	0.82 (0.80, 0.84)	5,475	139	0.94 (0.92, 0.97)	14,121	354	0.86 (0.85, 0.88)
WI	10,994	282	1.06 (1.04, 1.08)	6,704	172	1.17 (1.14, 1.20)	17,469	448	1.09 (1.08, 1.11)
Total	198,042	266		109,818	147		304,961	409	
Ratio of Highest/Lowest Rates		1.83			1.54			1.54	

**Table 3:** Correlations between total revascularization (PCI plus CABG) rates (per 100,000 age 20 and older) with state-level socio-economic status, cardiovascular surgery and cardiology work forces data, utilization of diagnostic catheterization, and measures of the burden of coronary heart diseases.

Correlating variables	Correlation Coefficient (R)	P-Value
Socio-Economic Status		
% of Population That is White	0.22	0.51
% of Population below Poverty Level	-0.03	0.94
Unemployment Rate (%)	-0.42	0.20
% of Population on Medicaid	-0.34	0.30
Cardiovascular Surgery and Cardiology Workforces		
No. of Cardiovascular Surgeons per 100,000 Population	0.64	0.03
No. of Cardiologists per 100,000 Population	0.22	0.51
No. of Interventional Cardiologists per 100,000 Population	0.25	0.46
No. of General Invasive Cardiologists per 100,000 Population	0.01	0.98
No. of Interventional Cardiologists Plus Cardiovascular Surgeons per 100,000 Population	0.38	0.25
Utilization of Diagnostic Catheterization		
No. of Diagnostic Catheterizations per 1,000 Medicare Enrollees	0.83	0.002
Measures of the Burden of Coronary Heart Diseases		
Age/Sex Adjusted Admissions/100,000 population for Acute Myocardial Infarction	0.46	0.15
Age/Sex Adjusted Admissions/100,000 population for Any Coronary Heart Diseases	0.64	0.03

**Table 4:** Proportion of PCI cases among revascularization (PCI or CABG) patients by state, 1999.

	1 Vessel Attempted			≥ 2 Vessels Attempted		
		
State	No. of Cases	Adjusted % of PCI*	Ratio to Mean (95% CI)	No. of Cases	Adjusted % of PCI*	Ratio to Mean (95% CI)
AZ	8,972	***94.0***	***0.97 (0.95, 0.99)***	6,029	25.9	1.16 (1.10, 1.12)
CA	42,265	97.3	1.01 (1.00, 1.02)	34,817	23.4	1.05 (1.03, 1.07)
CO	6,066	97.7	1.01 (0.98, 1.04)	3,673	23.1	1.04 (0.97, 1.11)
FL	40,507	97.4	1.01 (1.00, 1.02)	31,424	24.3	1.09 (1.06, 1.11)
IA	6,131	96.9	1.00 (0.98, 1.03)	4,473	***29.0***	***1.30 (1.23, 1.37)***
MD	8,450	***97.9***	***1.01 (0.99, 1.03)***	5,155	20.5	0.92 (0.86, 0.98)
NY	29,779	96.6	1.00 (0.99, 1.01)	21,179	19.3	0.87 (0.84, 0.89)
OR	4,344	95.5	0.99 (0.96, 1.02)	3,983	***10.4***	***0.46 (0.42, 0.51)***
SC	7,179	96.2	0.99 (0.97, 1.02)	5,511	18.3	0.82 (0.77, 0.87)
WA	7,681	96.2	0.99 (0.97, 1.02)	6,277	19.3	0.86 (0.82, 0.91)
WI	9,176	95.1	0.98 (0.96,1.00)	8,037	23.7	1.06 (1.02, 1.11)
Total	170,550	96.8		130,558	22.3	
Ratio of Highest/Lowest Proportions		1.04			2.80	

* Adjusted for age, sex, source of admission, acute myocardial infarction, and other comorbidities.
